# Supplementary material for: Optimizing Histatin 5: Effects of K13 and K17 Substitutions on Proteolytic Stability and Antifungal Activity
Source: Comput Struct Biotechnol J. 2026 Jul 14;35(1):0159. doi: 10.34133/csbj.0159 (PMC13365577; doi:10.34133/csbj.0159)
Supplement: Supplementary 1 — Figs. S1 to S6 Tables S1 to S3 [file csbj.0159.f1.pdf]

SUPPORTING INFORMATION

Optimizing histatin 5: Effects of K13 and K17 substitutions on proteolytic stability and antifungal activity

Wright K. Makambi[1], Victoria L. Chiu[1], Lydia Kasper[2], Bernhard Hube[2,3], and Amy J. Karlsson[1]

[1] Department of Chemical and Biomolecular Engineering, University of Maryland, College Park, USA
[2] Department of Microbial Pathogenicity Mechanisms, Leibniz Institute for Natural Product Research and Infection Biology – Hans Knöll Institute, Jena, Germany
[3]Institute of Microbiology, Friedrich-Schiller University, Jena, Germany

** Correspondence:*
Amy J. Karlsson
Department of Chemical and Biomolecular Engineering
University of Maryland
4418 Stadium Drive
College Park, MD 20742
Email: ajkarl@umd.edu
Phone: 301-405-2610

TABLE OF CONTENTS

Figure S1. Representative gel images for proteolysis of Hst5 and variants with modifications at K13 by Sap5, Sap6, and Sap10 .... Page 1

Figure S2. Representative gel images for proteolysis of Hst5 and variants with modifications at K17 by Sap5, Sap6, and Sap10 .... Page1

Figure S3. Representative gel images for proteolysis of Hst5 and variants with modifications at K13 by Sap1, Sap2, Sap3, and Sap9 .... Page 2

Figure S4. Representative gel images for proteolysis of Hst5 and variants with modifications at K17 by Sap1, Sap2, Sap3, and Sap9 .... Page 2

Table S1. *P*-values for data evaluating proteolysis of Hst5 and variants by Saps and saliva (Figures 1, 2, and 6) .... Page 3

Table S2. *P*-values for data evaluating antifungal activity of Hst5 and variants after incubation with and without Saps (Figures 3, 4, 5, and 7) .... Page 3

Figure S5. Gel images for proteolysis of Hst5 and variants with modification at K13 and K17 by saliva .... Page 4

Figure S6. Fragments produced by incubation of Hst5 and K17W with saliva. .... Page 5

Table S3. *P*-values for data evaluating the prevention of biofilm formation by Hst5 and K17W (Figure 8) .... Page 6

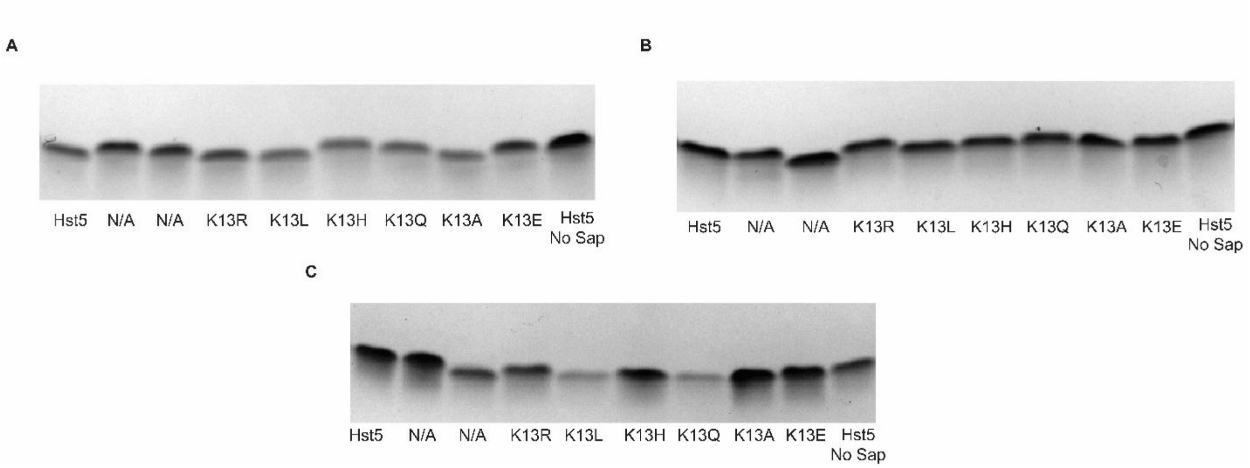

Figure S1. Representative gel images for proteolysis of Hst5 and variants with modifications at K13 by recombinant (A) Sap5, (B) Sap6, and (C) Sap10. Lanes marked “N/A” contain variants that are not relevant to this work.

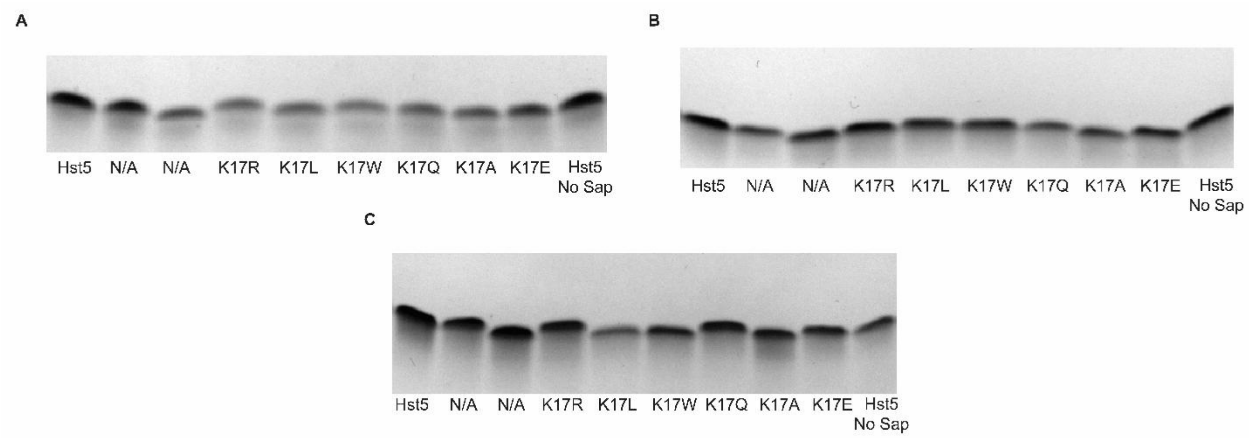

Figure S2. Representative gel images for proteolysis of Hst5 and variants with modifications at K17 by recombinant (A) Sap5, (B) Sap6, and (C) Sap10. Lanes marked “N/A” contain variants that are not relevant to this work.

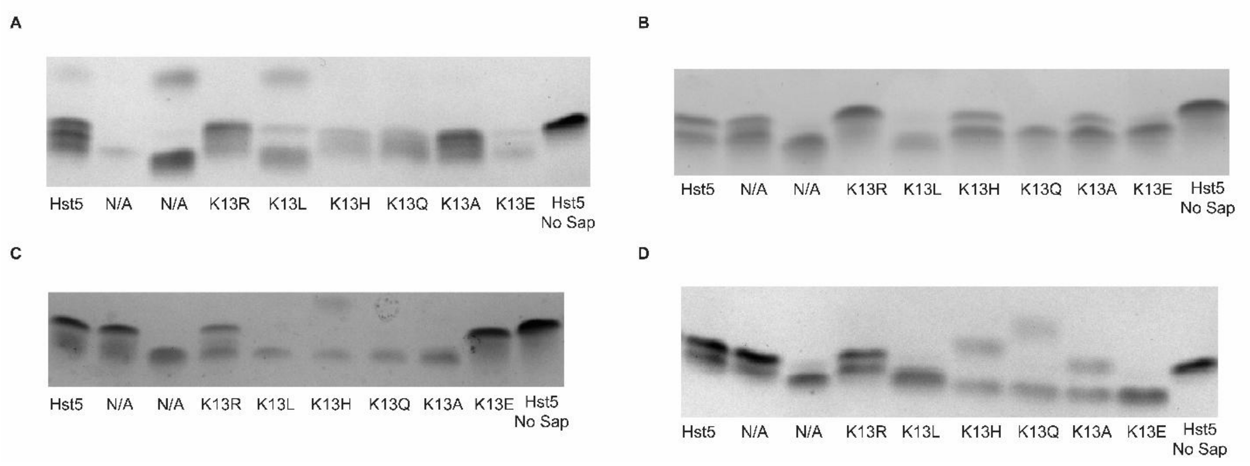

Figure S3. Representative gel images for proteolysis of Hst5 and variants with modifications at K13 by recombinant (A) Sap1, (B) Sap2, (C) Sap3, and (D) Sap9 Lanes marked “N/A” contain variants that are not relevant to this work.

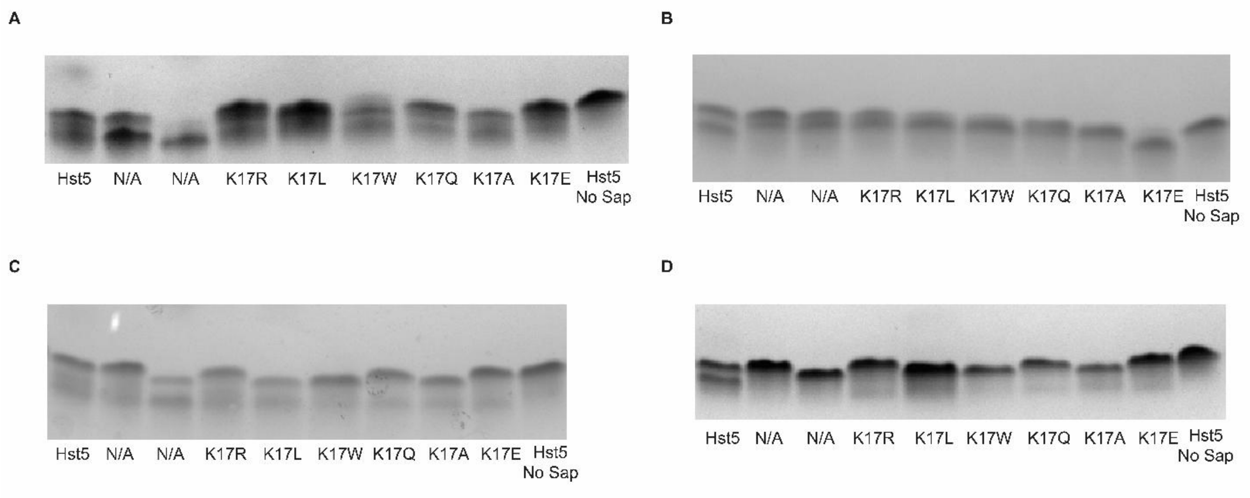

Figure S4. Representative gel images for proteolysis of Hst5 and variants with modifications at K17 by recombinant (A) Sap1, (B) Sap2, (C) Sap3, and (D) Sap9. Lanes marked “N/A” contain variants that are not relevant to this work.

Table S1. *P*-values for data evaluating proteolysis of Hst5 and variants by Saps and saliva (Figures 1, 2, and 6).

Peptide	*P*-values (Proteolysis assay)[a]				
	Sap1	Sap2	Sap3	Sap9	Saliva
K13R	0.9145	0.0286	0.9998	0.9877	0.0122
K13L	< 0.0001	< 0.0001	< 0.0001	< 0.0001	0.4414
K13H	0.0016	< 0.0001	< 0.0001	< 0.0001	0.8503
K13Q	0.0001	< 0.0001	< 0.0001	< 0.0001	0.9854
K13A	0.0079	< 0.0001	< 0.0001	0.946	0.0507
K13E	0.0013	< 0.0001	0.0008	< 0.0001	0.9614
K17R	0.059	0.010	1.000	< 0.0001	1.000
K17L	0.160	< 0.0001	0.940	< 0.0001	0.047
K17W	0.082	< 0.0001	< 0.0001	< 0.0001	0.049
K17Q	0.006	< 0.0001	0.003	< 0.0001	1.000
K17A	0.267	< 0.0001	0.033	< 0.0001	1.000
K17E	0.022	0.189	0.007	< 0.0001	0.412

[a] *P*-values for two-way ANOVA with α = .05 and Dunnett's multiple comparison tests (comparisons to degraded Hst5)

Table S2. *P*-values for data evaluating antifungal activity of Hst5 and variants after incubation with and without Saps (Figures 3, 4, 5 and 7).

Peptide	*P*-values (Antifungal activity assay)[a]					
	No Sap (4.7 µg/mL)[b]	Sap1 (4.7 µg/mL)	Sap2 (9.4 µg/mL)	Sap3 (18.8 µg/mL)	Sap9 (18.8 µg/mL)	Saliva (9.4 µg/mL)
K13R	0.0006	< 0.0001	0.9994	< 0.0001	0.0447	0.9997
K13L	0.9996	< 0.0001	< 0.0001	< 0.0001	< 0.0001	< 0.0001
K13H	0.1802	< 0.0001	< 0.0001	< 0.0001	< 0.0001	< 0.0001
K13Q	< 0.0001	< 0.0001	< 0.0001	< 0.0001	< 0.0001	< 0.0001
K13A	0.0073	< 0.0001	< 0.0001	< 0.0001	< 0.0001	< 0.0001
K13E	< 0.0001	< 0.0001	< 0.0001	< 0.0001	< 0.0001	< 0.0001
K17R	0.486	< 0.0001	0.067	0.914	< 0.0001	0.890
K17L	0.131	0.110	0.039	< 0.0001	< 0.0001	< 0.0001
K17W	0.240	< 0.0001	0.039	0.002	< 0.0001	< 0.0001
K17Q	0.000	< 0.0001	0.026	0.887	< 0.0001	< 0.0001
K17A	0.009	< 0.0001	0.020	0.971	< 0.0001	< 0.0001
K17E	< 0.0001	0.002	0.647	0.997	0.012	< 0.0001

[a] *P*-values for two-way ANOVA with α = 0.05 and Dunnett's multiple comparison tests (comparisons to degraded Hst5).
[b] Provided concentration is the concentration at which statistical analysis was performed.

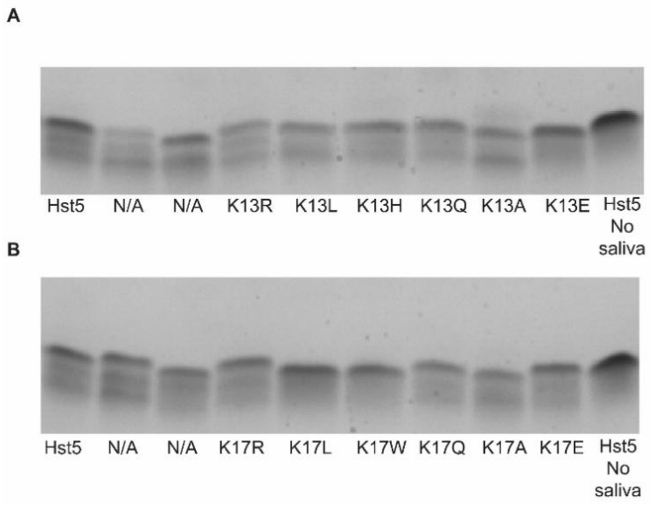

Figure S5. Gel images for proteolysis of Hst5 and variants with modification at (A) K13 and (B) K17 by saliva. Irrelevant lanes have been cropped out of the gel images. Lanes marked "N/A" contain variants that are not relevant to this work.

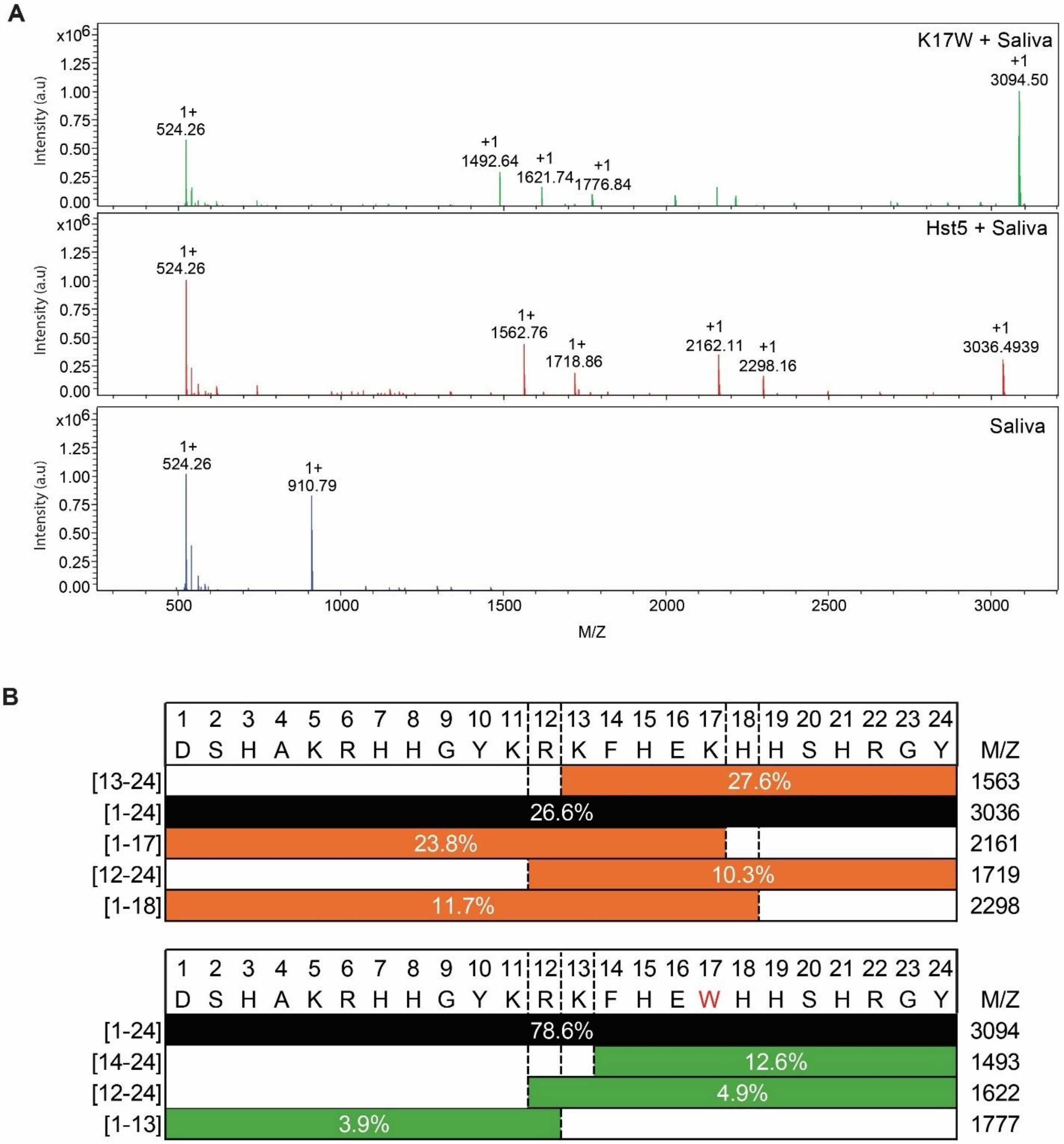

Figure S6. Fragments produced by incubation of Hst5 and K17W with saliva. Peptides were incubated with whole saliva for 2 h at 30 °C and analyzed by mass spectrometry. A) Deconvoluted mass spectra for saliva alone, Hst5 incubated with saliva, and K17W incubated with saliva. B) Identified peptide fragments generated by saliva-mediated degradation. Values within each block indicate the relative fraction of that fragment in the sample. Peaks not assigned to peptide fragments represent impurities or signals unrelated to Hst5 or K17W.

Table S3. *P*-values for data evaluating the prevention of biofilm formation by Hst5 and K17W (Figure 8).

Peptide	*P*-values (Biofilm prevention assay)[a]				
	12 µM	47 µM	94 µM	188 µM	750 µM
K17W	0.979	0.679	0.029	0.019	0.243

[a] *P*-values for two-way ANOVA with $\alpha = 0.05$ and Sidak multiple comparison test (comparison to Hst5).
